# Mechanistic prediction and validation of Brevilin A Therapeutic effects in Lung Cancer

**DOI:** 10.1186/s12906-024-04516-z

**Published:** 2024-06-05

**Authors:** Ruixue Wang, Cuiyun Gao, Meng Yu, Jialing Song, Zhenzhen Feng, Ruyu Wang, Huafeng Pan, Haimeng Liu, Wei Li, Xiangzhen Fan

**Affiliations:** 1 Shandong University of Traditional Chinese Medicine, Jinan, Shandong China; 2 Department of Rehabilitation Medicine, Binzhou Medical University Hospital, Binzhou, Shandong China; 3 School of Rehabilitation Medicine, Binzhou Medical University, Yantai, Shandong China; 4 School of clinical medicine, Jiangxi University of Chinese Medicine, Nanchang, Jiangxi China; 5 Science and Technology Innovation Center, Guangzhou University of Chinese Medicine, Guangzhou, Guangdong China

**Keywords:** Brevilin A, Lung cancer, Molecular mechanism, Network pharmacology, Molecular docking, Experimental validation

## Abstract

**Background:**

Traditional Chinese medicine (TCM) has been found widespread application in neoplasm treatment, yielding promising therapeutic candidates. Previous studies have revealed the anti-cancer properties of Brevilin A, a naturally occurring sesquiterpene lactone derived from Centipeda minima (L.) A.Br. (*C. minima*), a TCM herb, specifically against lung cancer. However, the underlying mechanisms of its effects remain elusive. This study employs network pharmacology and experimental analyses to unravel the molecular mechanisms of Brevilin A in lung cancer.

**Methods:**

The Batman-TCM, Swiss Target Prediction, Pharmmapper, SuperPred, and BindingDB databases were screened to identify Brevilin A targets. Lung cancer-related targets were sourced from GEO, Genecards, OMIM, TTD, and Drugbank databases. Utilizing Cytoscape software, a protein-protein interaction (PPI) network was established. Gene Ontology (GO), Kyoto Encyclopedia of Genes and Genomes (KEGG), Gene set enrichment analysis (GSEA), and gene-pathway correlation analysis were conducted using R software. To validate network pharmacology results, molecular docking, molecular dynamics simulations, and in vitro experiments were performed.

**Results:**

We identified 599 Brevilin A-associated targets and 3864 lung cancer-related targets, with 155 overlapping genes considered as candidate targets for Brevilin A against lung cancer. The PPI network highlighted STAT3, TNF, HIF1A, PTEN, ESR1, and MTOR as potential therapeutic targets. GO and KEGG analyses revealed 2893 enriched GO terms and 157 enriched KEGG pathways, including the PI3K-Akt signaling pathway, FoxO signaling pathway, and HIF-1 signaling pathway. GSEA demonstrated a close association between hub genes and lung cancer. Gene-pathway correlation analysis indicated significant associations between hub genes and the cellular response to hypoxia pathway. Molecular docking and dynamics simulations confirmed Brevilin A’s interaction with PTEN and HIF1A, respectively. In vitro experiments demonstrated Brevilin A-induced dose- and time-dependent cell death in A549 cells. Notably, Brevilin A treatment significantly reduced HIF-1α mRNA expression while increasing PTEN mRNA levels.

**Conclusions:**

This study demonstrates that Brevilin A exerts anti-cancer effects in treating lung cancer through a multi-target and multi-pathway manner, with the HIF pathway potentially being involved. These results lay a theoretical foundation for the prospective clinical application of Brevilin A.

## Introduction

Lung cancer is a common malignancy with high incidence and mortality worldwide [1]. Non-small cell lung cancer (NSCLC) accounts for approximately 80–85% of all lung cancer cases [2]. Despite considerable advancements in the prevention, early diagnosis, and treatment of NSCLC, the clinical outcomes for advanced NSCLC remain suboptimal [3, 4]. Therefore, there exists an imperative requirement to innovate novel therapeutic strategies for the effective treatment of NSCLC [5].

Traditional Chinese medicine (TCM) has garnered attention as a promising avenue in cancer treatment, attributed to its commendable therapeutic efficacy and minimal side effects [6]. TCM products have demonstrated anticancer effects through diverse pathways and mechanisms [7]. Brevilin A, a sesquiterpene lactone derived from *C. minima*, exhibits a spectrum of pharmacological activities encompassing anti-cancer [8, 9], anti-oxidative [10], anti-inflammatory [11, 12], and immune-enhancing effects [13]. Previous studies have shown the potential of Brevilin A in combating various human malignancies, including lung cancer [14], nasopharyngeal carcinoma [15], multiple myeloma [16], gastric cancer [17], breast cancer [18], and prostate cancer [19]. A published study delineated that Brevilin A induces apoptosis in lung cancer cells by promoting reactive oxygen species (ROS) generation and inhibiting STAT3 activation [20]. However, the precise molecular mechanisms underlying Brevilin A’s action against lung cancer remain elusive. Moreover, Brevilin A exhibits a favorable pharmacokinetic profile and remarkable bioavailability, with no discernible acute toxicity observed in mice administered a substantial dosage of Brevilin A [16]. This implies the safety of Brevilin A, thereby encouraging further exploration of its therapeutic potential in the context of lung cancer.

**Fig. 1:**
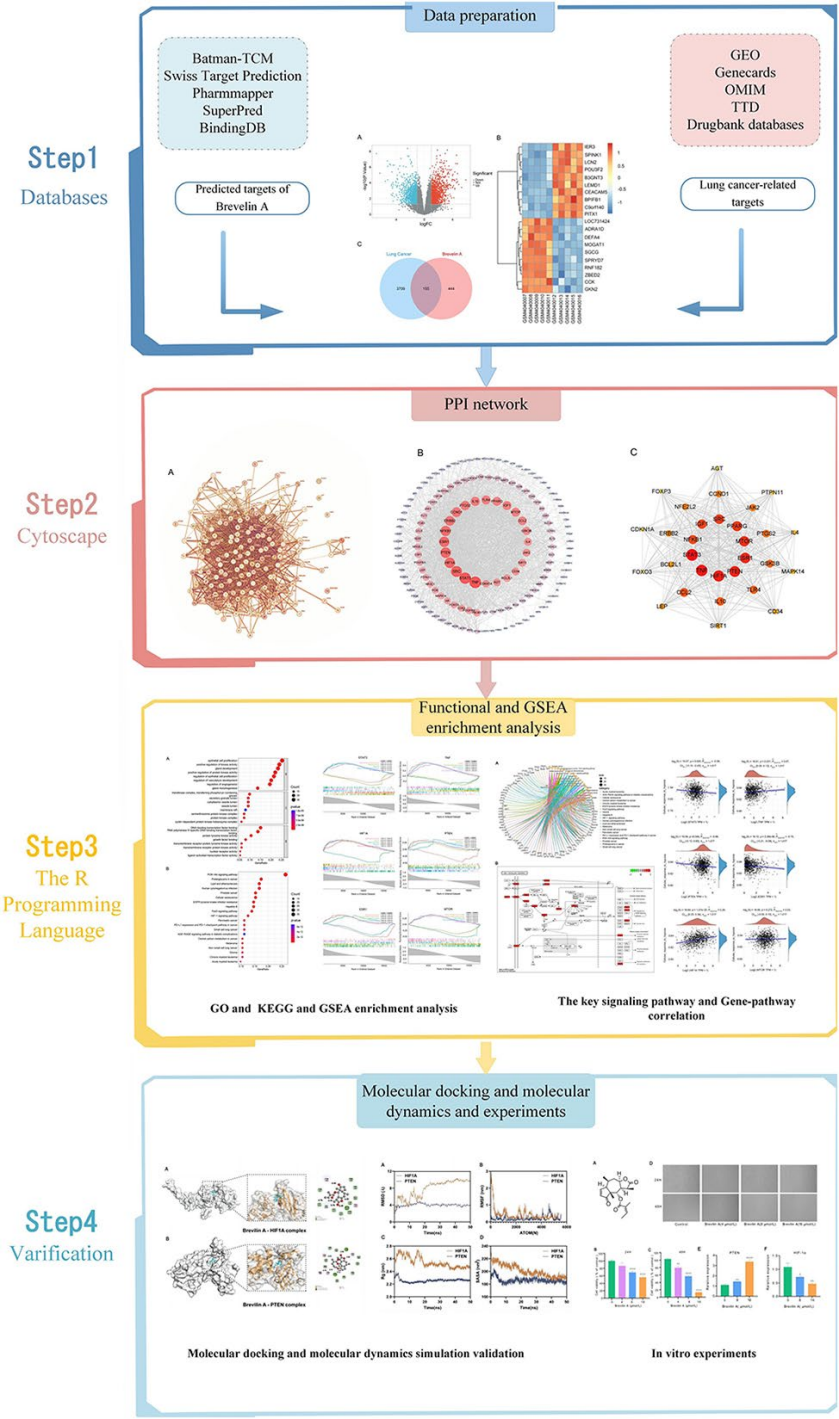
Mechanistic insights into Brevilin A action against lung cancer. Schematic diagram summarizing the mechanisms underlying Brevilin A action against lung cancer using network pharmacology, molecular docking, and experimental validation

Network pharmacology is a robust bioinformatics tool for comprehensively identifying candidate targets, functions, and mechanisms of TCM in disease treatment [21]. In this study, we employed network pharmacology to predict the potential mechanisms of Brevilin A in lung cancer. Molecular docking and in vitro experiments were conducted to validate the obtained results. The workflow of this study is elucidated in Fig. 1.

## Materials and methods

### Retrieval of potential targets of Brevilin A

The identification of targets associated with the compound denoted as “Brevilin A” was accomplished through the application of the Bioinformatics Analysis Tool for the Molecular Mechanism of Traditional Chinese Medicine database, herein referred to as Batman-TCM (http://bionet.ncpsb.org.cn/batman-tcm/index.php), whereby targets achieving a score exceeding 5 were considered. The 3D structures and Isomeric SMILES of the aforementioned compound, Brevilin A, were procured from the PubChem database (https://pubchem.ncbi.nlm.nih.gov/). Utilizing the structural information of Brevilin A, prospective targets were elucidated through computational analyses conducted on multiple platforms, namely Swiss Target Prediction (http://swisstargetprediction.ch/), Pharmmapper (http://lilab-ecust.cn/pharmmapper/index.html), SuperPred (https://prediction.charite.de/), and BindingDB (https://www.bindingdb.org/bind/index.jsp) databases. The resultant target predictions underwent standardization procedures utilizing the UniProt database.

### Screening for lung cancer-related targets

The keyword “Lung cancer” guided our exploration in the Gene Expression Database (GEO, https://www.ncbi.nlm.nih.gov/geo/), resulting in the retrieval of the GSE136043 dataset which contains mRNA lung tissue microarray data from five patients with lung cancer and five healthy volunteers. Gene differential expressions (DEGs) in the GSE136043 dataset were then analyzed using the R package limma, wherein genes exhibiting log2 (fold change) > 1 or < -1, coupled with a p-value < 0.05, were designated as differentially expressed. Concurrently, targets associated with lung cancer were ascertained through a comprehensive inquiry encompassing the Omim (https://www.omim.org/), GeneCards (https://www.genecards.org/), TTD (https://db.idrblab.net/ttd/), and Drugbank (https://go.drugbank.com/) databases. The outcomes from these diverse databases were amalgamated, and duplicates were expunged, and the resulting targets underwent standardization employing the UniProt database. The identification of candidate targets linked to both the pharmaceutical agent denoted as “Brevilin A” and lung cancer was achieved through exploration of the Xiantao Academic website (https://www.xiantaozi.com).

### Construction of protein-protein interaction (PPI) network

To ascertain information regarding interactions among proteins, data pertaining to candidate target genes were submitted to the esteemed STRING database (https://string-db.org/) [22]. The designated species for analysis was “Homo sapiens,” and a minimum interaction score threshold of 0.400, denoting medium confidence, was applied. The outcomes were obtained in TSV format and imported into Cytoscape 3.8.0 for the purpose of visualizing the PPI network interactions. The utilization of the exceptional CytoHubba Cytoscape plugin facilitated the identification of pivotal genes within the PPI network. The Maximum Correlation Clique (MCC) for each node in the PPI network was calculated using the CytoHubba plugin, wherein larger and darker nodes indicated higher-scoring genes.

### GO and KEGG pathway enrichment analysis

The “clusterProfiler” package in R 4.3.1 software was employed to perform Gene Ontology (GO) and Kyoto Encyclopedia of Genes and Genomes (KEGG) enrichment analyses, utilizing a P-value<0.05 and Q-value<1 as the criteria for selection. The GO enrichment analysis encompassed cellular components (CC), biological processes (BP), and molecular functions (MF). The eight most notable terms for each category have been delineated, and bubble diagrams were generated using the R 4.3.1 software. The KEGG enrichment analysis aimed to elucidate the potential mechanisms by which Brevilin A engages with lung cancer. Subsequently, bubble charts were generated to visually represent the top 20 significant pathways, employing the R 4.3.1 software.

### GSEA enrichment analysis

To ascertain the association between key targets and potential mechanisms in lung cancer, Gene Set Enrichment Analysis (GSEA) was performed on the GSE136043 dataset using the R package “clusterProfiler,” with a P-value < 0.05 employed as the filtering criterion.

### Component-target molecular docking and molecular dynamics simulation

Molecular docking and molecular dynamics simulation represent computational techniques frequently utilized for the preliminary investigation of mechanisms and drug discovery. Their efficacy lies in their capacity to predict potential binding orientations and affinities of protein-ligand complexes [23, 24].

#### Molecular docking

In the preparatory phase, protein crystal structures were initially acquired from the Protein Data Bank (PDB, https://www.rcsb.org/ (accessed on 12 September 2023)). Subsequently, homology modeling was employed for the reconstruction of missing residue structures, utilizing a previously established template with reference to the SWISS-MODEL website [25]. The molecular structure of Brevilin A was obtained from the PubChem database (https://pubchem.ncbi.nlm.nih.gov/ (accessed on 12 September 2023)). Subsequent to acquisition, the molecular structure underwent geometry optimization employing the B3LYP approach and the 6-311 + + G (d, p) basis set, utilizing Gaussian 09 W and GaussView 5.0 software. The standard restrained electrostatic potential (RESP) of Brevilin A was calculated and applied using the Multiwfn program [26]. Molecular docking was performed on the SwissDock website, employing default settings [27]. The SwissDock website generated output clusters from each docking run. These clusters were then prioritized based on the FullFitness (FF) scoring function, a specific algorithm integrated into SwissDock. Subsequently, the individual conformers within each cluster were ranked by their FF scores, enabling us to select the conformer with the most favorable FF score for further assessment. The resultant docking sites between the ligand and the protein were visualized using PyMOL and Discovery Studio 2019 software [28].

#### Molecular dynamics simulation

The optimal conformations derived from molecular docking underwent comprehensive evaluation of binding stability through molecular dynamics simulation, utilizing Gromacs 2020.06 software [29]. The simulations employed the AMBER99SB-ILDN/GAFF force field, and the initial systems were established in a cubic box featuring a 1.0 nm layer, populated with the TIP3P water model. Energy minimizations were performed using the steepest descent algorithm. Subsequently, the systems were equilibrated with the canonical (NVT) and isothermal-isobaric (NPT) ensembles for 100 ps prior to the commencement of the molecular dynamics simulation. The equilibrium system was configured to maintain a temperature of 310 K and a standard pressure of 1.0 bar. The ensuing molecular dynamics simulations spanned a duration of 50 ns to evaluate the stability of the complex. Trajectory files were employed to calculate the root mean square deviation (RMSD), root mean square fluctuation (RMSF), Radius of gyration (Rg) value, and solvent accessible surface area (SASA). These parameters were selected for their capacity to offer insights into the structural states of the complex. To ascertain the binding free energies (BFE) of the complex, the molecular mechanics/Poisson-Boltzmann surface area (MM/PBSA) approach was applied. The BFE in an aqueous solvent (ΔGbind) is typically expressed as the sum of three components: (1) ΔE_MM_, signifying the change in gas-phase molecular mechanics energy; (2) ΔG_PB_, indicating the change in polar solvation energy; and (3) ΔG_SA_, denoting the change in non-polar solvation energy. Additionally, the alteration in conformational entropy (–TΔS) was estimated using the interaction entropy (IE) method [30]. These calculations were performed using trajectory files at 1 ns intervals for the final 20 ns, during which the RMSD remained stable.

### Gene-pathway correlation analysis

RNA-sequencing expression profiles (level 3) and pertinent clinical data for lung cancer were acquired from the TCGA dataset (https://portal.gdc.com). Analysis was conducted utilizing the GSVA package in R software, with the parameter method=’ssgsea’ being chosen. The examination of the relationship between genes and pathway scores was carried out using Spearman correlation. All analytical procedures and R packages were implemented using R version 4.3.1. A p-value less than 0.05 was considered statistically significant.

### Experimental validation

#### Chemicals and reagents

Brevilin A with 99.71% purity and Cell Counting Kit-8 (CCK-8) kit were obtained from MCE (MedChemExpress, shanghai, China).

#### Cell culture

A549 cells were procured from the American Type Culture Collection (ATCC, Manassas, VA, USA) and subsequently cultivated in Dulbecco’s Modified Eagle Medium (DMEM) supplemented with 10% fetal bovine serum (FBS). All aforementioned reagents were acquired from Gibco (CA, USA). The cells were maintained in a humidified incubator with 5% CO2 at a temperature of 37℃.

#### Cell viability assay

A549 cells were cultured in 96-well plates with a density of 4000 cells per well. After 24 h of incubation, the cells were exposed to Brevilin A at specified concentrations for 24 and 48 h. Subsequently, the cells were treated with CCK-8 solution for an additional hour at 37℃, and the absorbance was quantified at 450 nm using a microplate reader (ThermoFisher, Waltham, MA).

#### Quantitative RT-PCR (qRT-PCR)

RNA was isolated from cells using the NucleoSpin RNA isolation Kit (Macherey-Nagel, Düren, Germany) and TRIzol™ reagent, respectively. Subsequently, reverse transcription-PCR was conducted using the RevertAid First Strand cDNA Synthesis Kit (Thermo Scientific, Waltham, USA) following the manufacturer’s instructions. Quantitative PCR (qPCR) analysis was performed on the ABI 7500 Fast Real-time PCR System using the Taq pro Universal SYBR qPCR Master Mix (Vazyme Biotech, Nanjing, China). Relative gene expression was determined using the ΔΔCt method, and the primer sequences are available upon request.

### Statistical analysis

Statistical analyses were conducted using GraphPad Prism 8.0. The data presented in this study are derived from a minimum of three independent experiments and are expressed as the mean ± standard error of the mean (SEM). To assess differences, unpaired t-tests and one-way analysis of variance (ANOVA) were employed. P-values less than 0.05 were deemed statistically significant.

## Results

### PPI network analyses

**Fig. 2:**
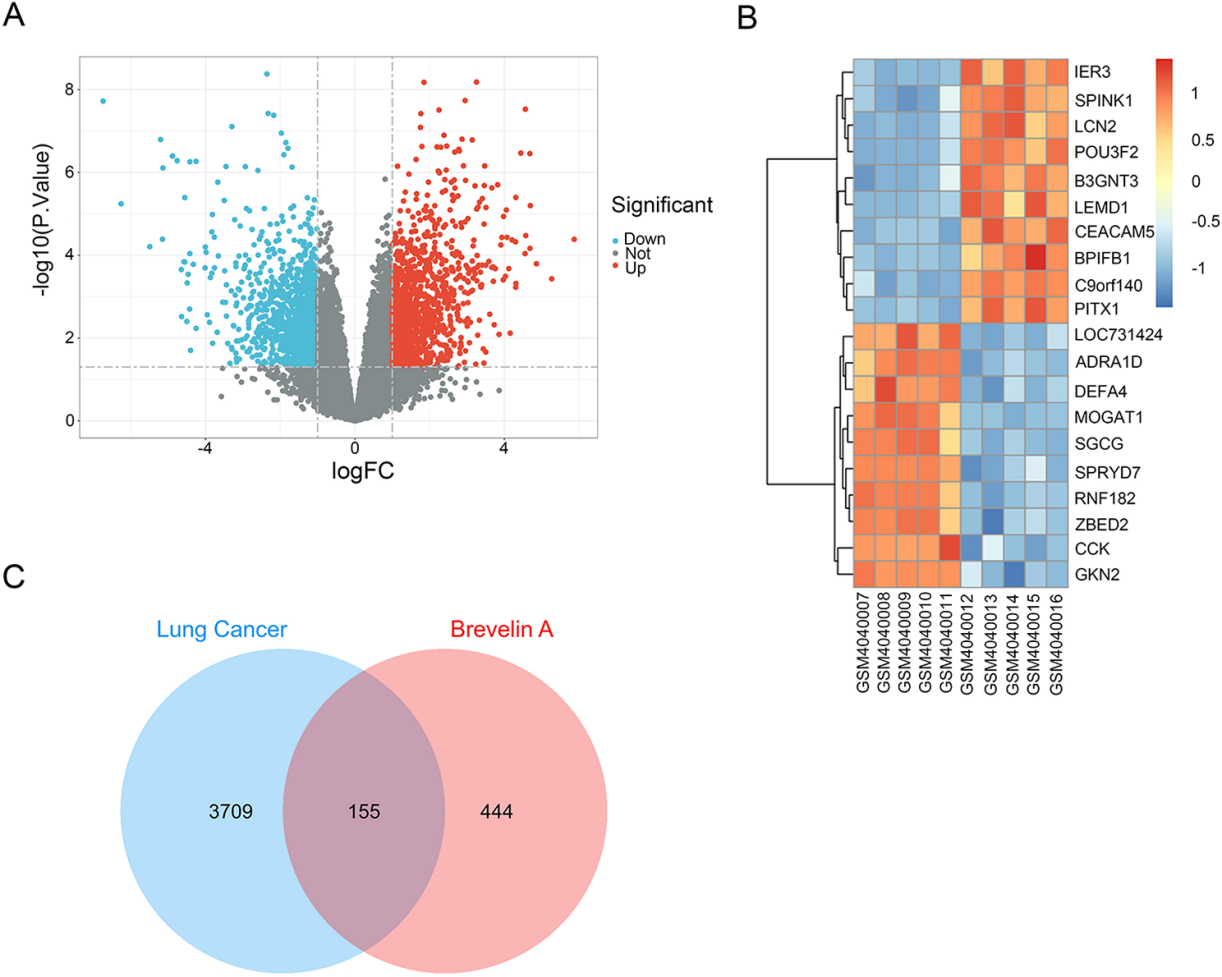
Differential gene expression analysis. **(A)** GEO Volcano Map and **(B)** GEO heatmap of the top 10 up- and down-regulated genes expression. Red and blue dots indicate up-regulated and down-regulated genes, respectively. **(C)** Venn diagram showing the overlap of Brevilin A-associated targets and lung cancer-related genes

**Fig. 3:**
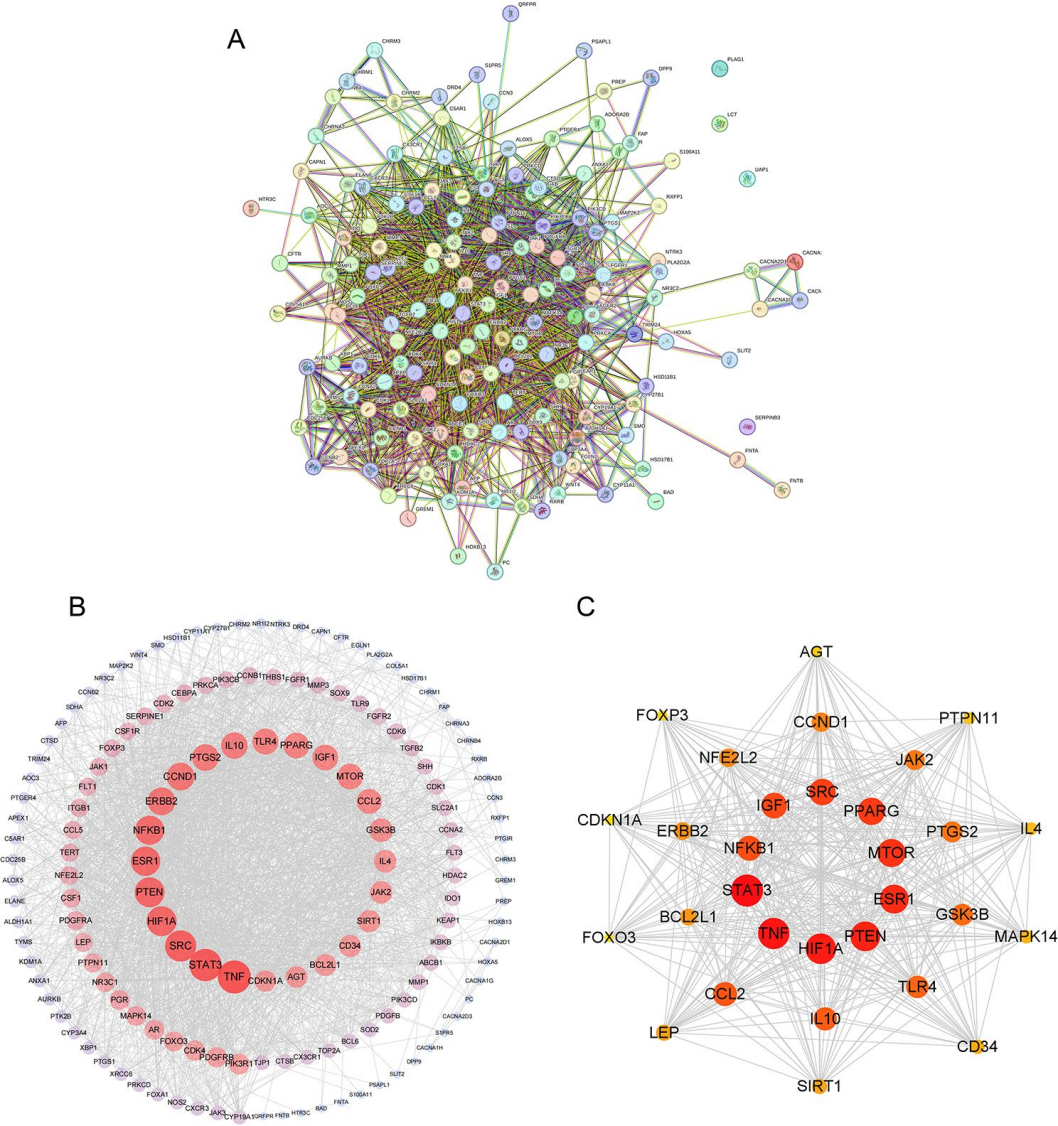
Protein-protein interaction (PPI) network analysis. **(A)** The PPI network. **(B)** Interaction between these genes. **(C)** Hub genes identified using the MCC method

A total of 2554 DEGs from GSE136043 dataset, comprising 1214 upregulated and 1340 downregulated genes, were identified and visually represented using a volcano plot (Fig. 2A) and a heatmap of the top 10 up- and down-regulated genes expression(Fig. 2B). Subsequently, we gathered 599 Brevilin A-related targets and 3864 lung cancer-related targets, resulting in 155 overlapping genes selected as Brevilin A candidate targets against lung cancer (Fig. 2C). A PPI network of the 155 overlapping targets was constructed, consisting of 151 nodes and 2134 edges (Fig. 3A, B**)**. Nodes with higher degrees were considered more pivotal in the network. The top 30 genes, exhibiting the highest degree of connectivity, are presented in Table 1. Identified through MCC scores, STAT3, TNF, HIF1A, PTEN, ESR1, and MTOR were identified as potential hub genes (Fig. 3C).

**Table 1:** Degree of hub regulatory genes analyzed by Cytoscape

Name	Symbol	Average shortest path length	Betweenness centrality	Closeness centrality	Degree	Neighborhood connectivity	MCC scores
Tumor necrosis factor	TNF	1.393333333	0.051584339	0.717703349	95	38.15789474	1.83541E + 18
Signal transducer and activator of transcription 3	STAT3	1.413333333	0.038666898	0.70754717	92	40.08695652	1.83542E + 18
Proto-oncogene tyrosine-protein kinase Src	SRC	1.44	0.070315983	0.694444444	88	38.20454545	1.82361E + 18
Phosphatidylinositol 3,4,5-trisphosphate 3-phosphatase and dual-specificity protein phosphatase PTEN	PTEN	1.473333333	0.03764339	0.678733032	83	40.53012048	1.83377E + 18
Hypoxia-inducible factor 1-alpha	HIF1A	1.48	0.036424015	0.675675676	83	41.46987952	1.83541E + 18
Estrogen receptor	ESR1	1.493333333	0.043621748	0.669642857	81	40.01234568	1.83294E + 18
Nuclear factor NF-kappa-B p105 subunit	NFKB1	1.506666667	0.032043207	0.663716814	80	41.475	1.80986E + 18
G1/S-specific cyclin-D1	CCND1	1.56	0.023045173	0.641025641	75	42.12	1.39526E + 18
Receptor tyrosine-protein kinase erbB-2	ERBB2	1.546666667	0.020795759	0.646551724	75	42.22666667	1.11099E + 18
Prostaglandin G/H synthase 2	PTGS2	1.566666667	0.02182886	0.638297872	71	43	1.4762E + 18
Interleukin-10	IL10	1.573333333	0.019330865	0.63559322	70	41.94285714	1.71236E + 18
Toll-like receptor 4	TLR4	1.586666667	0.016253195	0.630252101	68	42.35294118	1.69698E + 18
Serine/threonine-protein kinase mTOR	MTOR	1.58	0.045501984	0.632911392	67	44.37313433	1.83175E + 18
Peroxisome proliferator-activated receptor gamma	PPARG	1.586666667	0.028081306	0.630252101	67	43.86567164	1.82687E + 18
Insulin-like growth factor I	IGF1	1.58	0.021387304	0.632911392	67	45.85074627	1.81178E + 18
C-C motif chemokine 2	CCL2	1.613333333	0.013568449	0.619834711	64	44.03125	1.71757E + 18
Glycogen synthase kinase-3 beta	GSK3B	1.64	0.013339113	0.609756098	59	45.84745763	1.49879E + 18
Interleukin-4	IL4	1.686666667	0.007190134	0.592885375	55	45.72727273	3.11169E + 17
Tyrosine-protein kinase JAK2	JAK2	1.666666667	0.023161161	0.6	55	47.58181818	1.46422E + 18
NAD-dependent protein deacetylase sirtuin-1	SIRT1	1.706666667	0.006113591	0.5859375	54	47.37037037	7.23847E + 17
Hematopoietic progenitor cell antigen CD34	CD34	1.72	0.010031337	0.581395349	53	46.86792453	6.5861E + 17
Bcl-2-like protein 1	BCL2L1	1.706666667	0.009762444	0.5859375	53	48.66037736	1.05405E + 18
Angiotensinogen	AGT	1.686666667	0.061142799	0.592885375	52	40.94230769	2.57726E + 17
Cyclin-dependent kinase inhibitor 1	CDKN1A	1.766666667	0.017783501	0.566037736	51	45.52941176	1.16052E + 17
Phosphatidylinositol 3-kinase regulatory subunit alpha	PIK3R1	1.733333333	0.007475376	0.576923077	49	44.6122449	7.11254E + 12
Platelet-derived growth factor receptor beta	PDGFRB	1.733333333	0.010130617	0.576923077	47	48.57446809	2.18903E + 16
Cyclin-dependent kinase 4	CDK4	1.78	0.005998832	0.561797753	47	47.68085106	5.29473E + 16
Forkhead box protein O3	FOXO3	1.766666667	0.004839212	0.566037736	46	48.73913043	9.43103E + 16
Mitogen-activated protein kinase 14	MAPK14	1.76	0.005257046	0.568181818	45	50.75555556	3.24618E + 17
Progesterone receptor	PGR	1.78	0.013641332	0.561797753	45	42.68888889	2.21373E + 14

### GO and KEGG function enrichment analysis

**Fig. 4:**
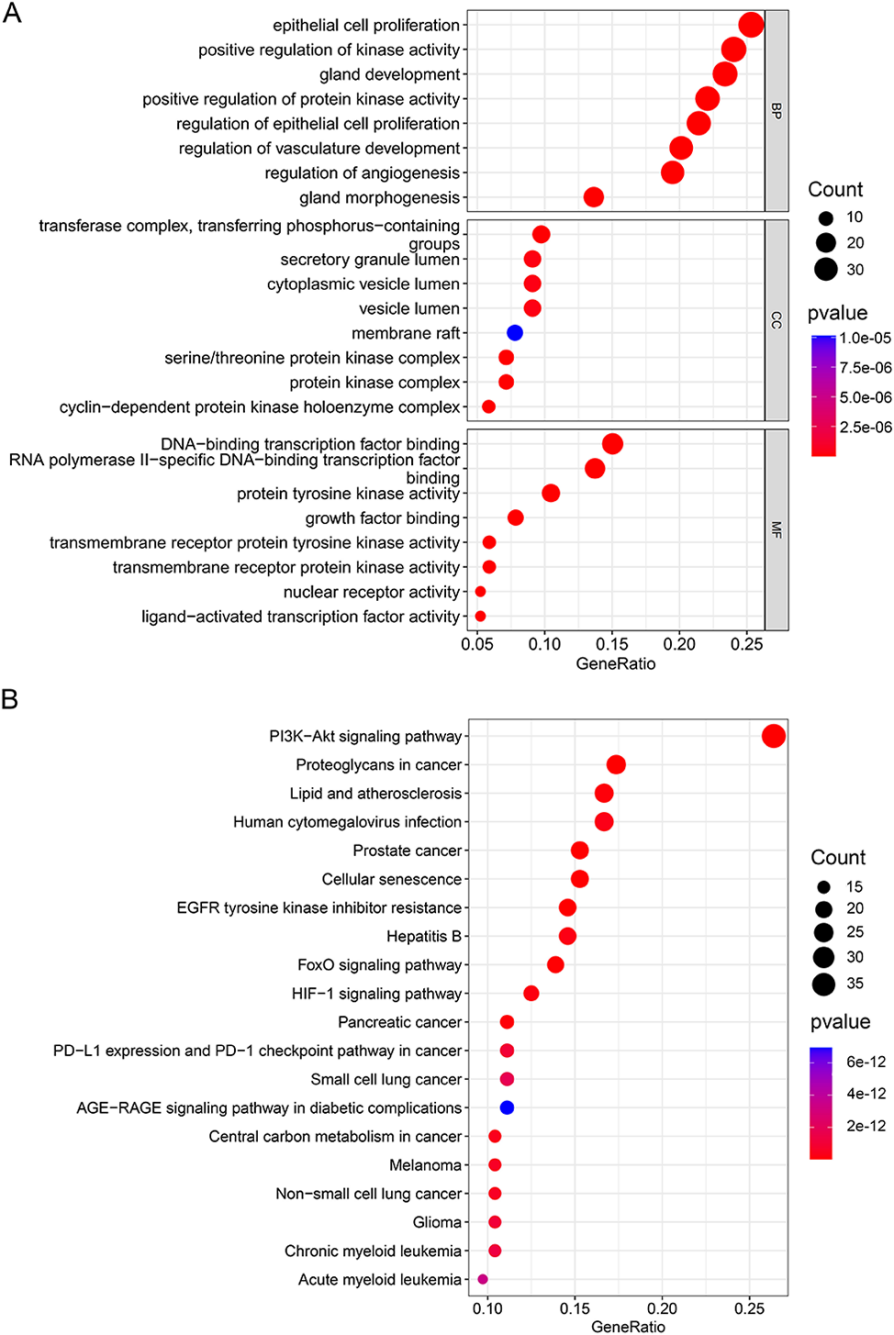
Functional enrichment analysis. Bubble chart of GO **(A)** and KEGG **(B)** function enrichment analysis

**Fig. 5:**
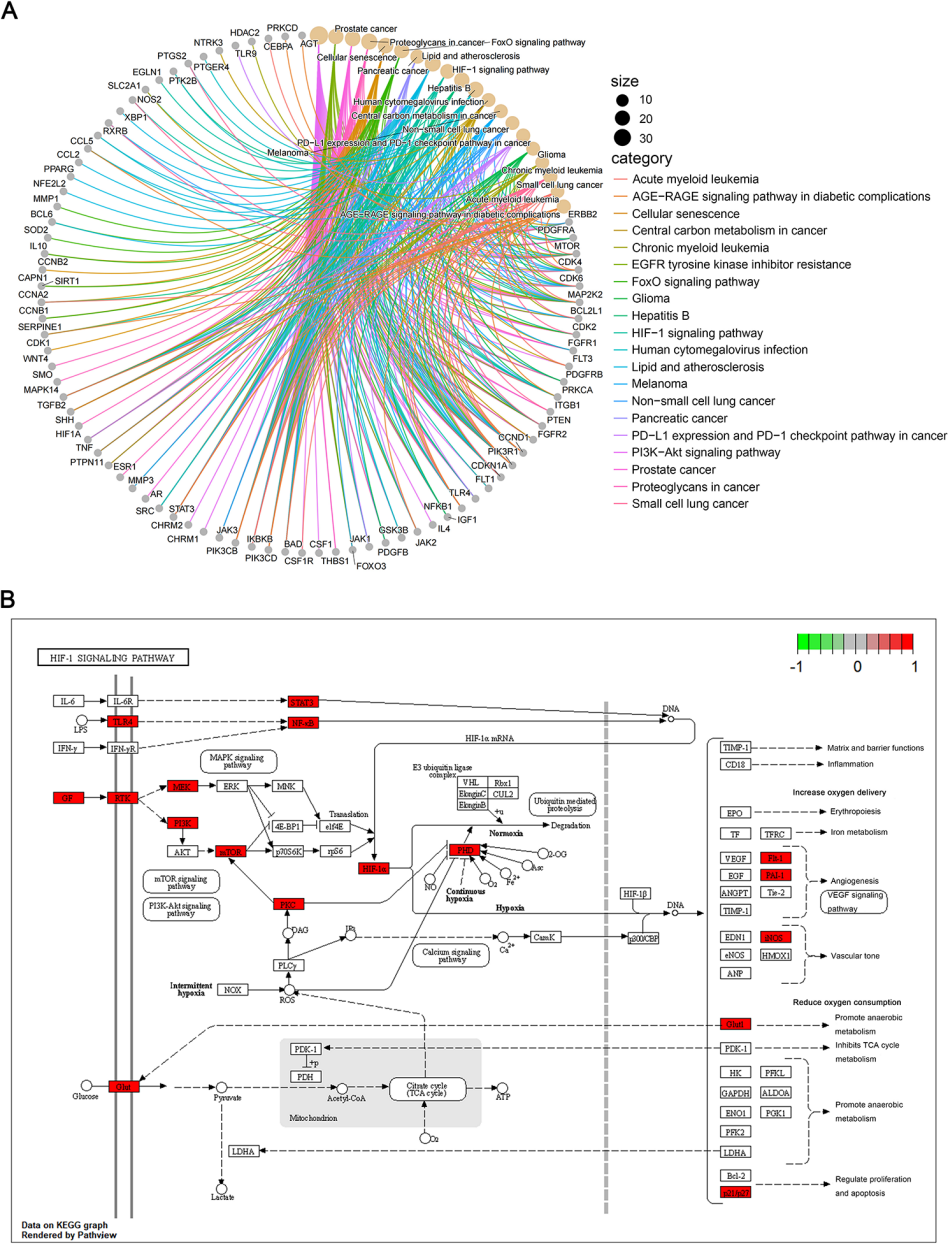
Brevilin A target pathway in lung cancer. **(A)** Brevilin A target-major pathway-lung cancer. **(B)** Distribution of key targets in the HIF-1 signaling pathway

We conducted GO and KEGG function enrichment analyses on the 155 overlapping targets, resulting in the identification of 2893 GO terms and 157 KEGG pathways. The top 8 significant GO terms from each category are depicted in Fig. 4A. In the CC category, enrichments were observed in transferase complexes facilitating the transfer of phosphorus-containing groups, secretory granule lumen, and cytoplasmic vesicle lumen. In the BP category, enrichments included epithelial cell proliferation, positive regulation of kinase activity, and gland development. The MF category exhibited enrichments in DNA-binding transcription factor binding, RNA polymerase II-specific DNA-binding transcription factor binding, and protein tyrosine kinase activity (Fig. 4A). The top 20 significant pathways, such as the PI3K-Akt signaling pathway, FoxO signaling pathway, and HIF-1 signaling pathway, are presented in Fig. 4B; Table 2. A chord diagram was employed to visually depict the relationship between enriched KEGG pathways and genes (Fig. 5A**)**, while Fig. 5B illustrates the distribution of key targets in the HIF-1 signaling pathway.

**Table 2:** KEGG pathway enrichment analysis

ID	Description	*P* value	*P*.adjust	Q value	Count
hsa04151	PI3K-Akt signaling pathway	1.05E-20	2.69E-18	1.09E-18	38
hsa01521	EGFR tyrosine kinase inhibitor resistance	3.92E-20	5.02E-18	2.04E-18	21
hsa05215	Prostate cancer	2.08E-19	1.77E-17	7.21E-18	22
hsa05205	Proteoglycans in cancer	3.62E-15	2.32E-13	9.44E-14	25
hsa04218	Cellular senescence	8.86E-15	4.54E-13	1.85E-13	22
hsa04068	FoxO signaling pathway	3.41E-14	1.45E-12	5.91E-13	20
hsa05212	Pancreatic cancer	7.86E-14	2.88E-12	1.17E-12	16
hsa05417	Lipid and atherosclerosis	1.01E-13	3.24E-12	1.32E-12	24
hsa04066	HIF-1 signaling pathway	1.75E-13	4.97E-12	2.02E-12	18
hsa05161	Hepatitis B	2.10E-13	5.38E-12	2.19E-12	21
hsa05163	Human cytomegalovirus infection	2.79E-13	6.50E-12	2.64E-12	24
hsa05230	Central carbon metabolism in cancer	3.72E-13	7.94E-12	3.23E-12	15
hsa05218	Melanoma	5.79E-13	1.06E-11	4.31E-12	15
hsa05223	Non-small cell lung cancer	5.79E-13	1.06E-11	4.31E-12	15
hsa05235	PD-L1 expression and PD-1 checkpoint pathway in cancer	1.07E-12	1.75E-11	7.13E-12	16
hsa05214	Glioma	1.10E-12	1.75E-11	7.13E-12	15
hsa05220	Chronic myeloid leukemia	1.35E-12	2.03E-11	8.25E-12	15
hsa05222	Small cell lung cancer	1.82E-12	2.59E-11	1.06E-11	16
hsa05221	Acute myeloid leukemia	3.41E-12	4.60E-11	1.87E-11	14
hsa04933	AGE-RAGE signaling pathway in diabetic complications	6.94E-12	8.89E-11	3.62E-11	16

### GSEA enrichment analysis

**Fig. 6:**
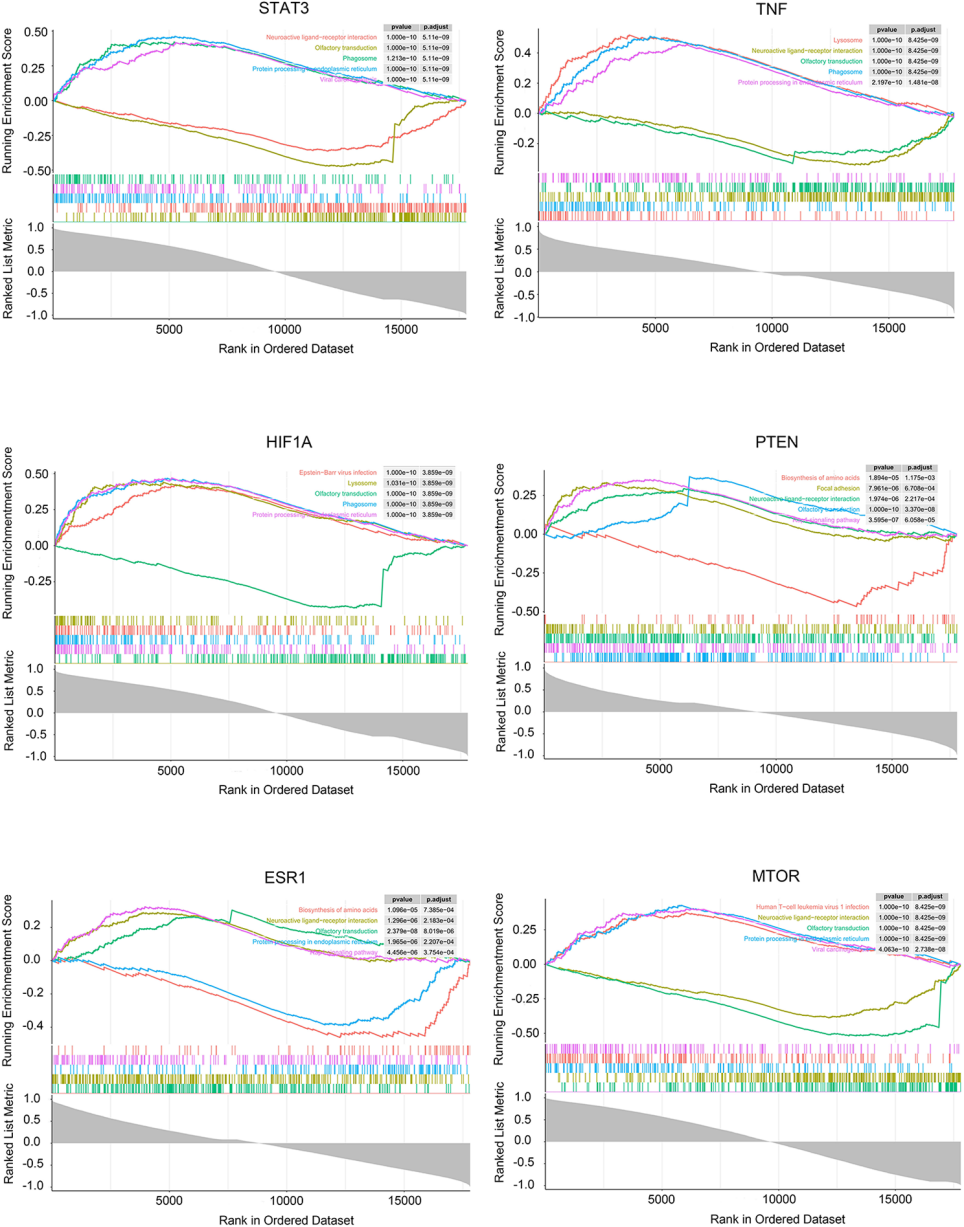
GSEA enrichment plot of hub genes

**Fig. 7:**
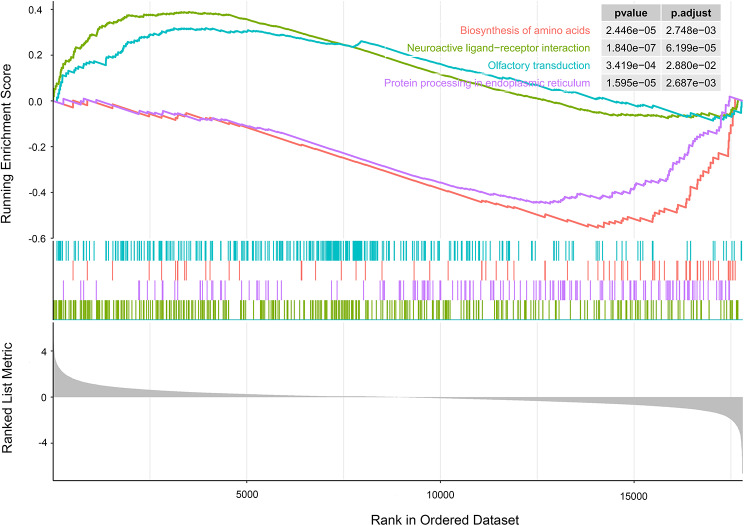
GSEA enrichment plot of GSE136043 dataset

To further elucidate the pathway analysis of DEGs, GSEA analysis was performed on both low and high expression of hub genes. The GSEA results are shown in Fig. 6, revealing that signaling pathways associated with the high expression phenotype of STAT3 encompass phagosome, protein processing in the endoplasmic reticulum, and viral carcinogenesis. Conversely, pathways linked to the low expression of STAT3 include neuroactive ligand − receptor interaction and olfactory transduction (Fig. 6A). For TNF, the high expression phenotype is correlated with pathways such as phagosome, protein processing in the endoplasmic reticulum, and lysosome, while the low expression is associated with neuroactive ligand − receptor interaction and olfactory transduction (Fig. 6B). HIF1A’s high expression phenotype is linked to Epstein − Barr virus infection, lysosome, phagosome, and protein processing in the endoplasmic reticulum, while its low expression is tied to olfactory transduction (Fig. 6C). The pathways associated with high PTEN expression include focal adhesion, neuroactive ligand − receptor interaction, olfactory transduction, and the rap1 signaling pathway, while low PTEN expression is connected to the biosynthesis of amino acids (Fig. 6D). ESR1’s high expression is associated with neuroactive ligand − receptor interaction, olfactory transduction, and the rap1 signaling pathway, while low ESR1 expression is linked to the biosynthesis of amino acids and protein processing in the endoplasmic reticulum (Fig. 6E). MTOR’s high expression is connected to human T − cell leukemia virus 1 infection, protein processing in the endoplasmic reticulum, and viral carcinogenesis, whereas low MTOR expression is associated with neuroactive ligand − receptor interaction and olfactory transduction (Fig. 6F). These results underscore that the significantly enriched pathways associated with core targets align closely with those implicated in lung cancer. Notably, biosynthesis of amino acids, neuroactive ligand − receptor interaction, olfactory transduction, and protein processing in the endoplasmic reticulum emerge as pathways intricately associated with lung cancer (Fig. 7).

### Gene-pathway correlation analysis

**Fig. 8:**
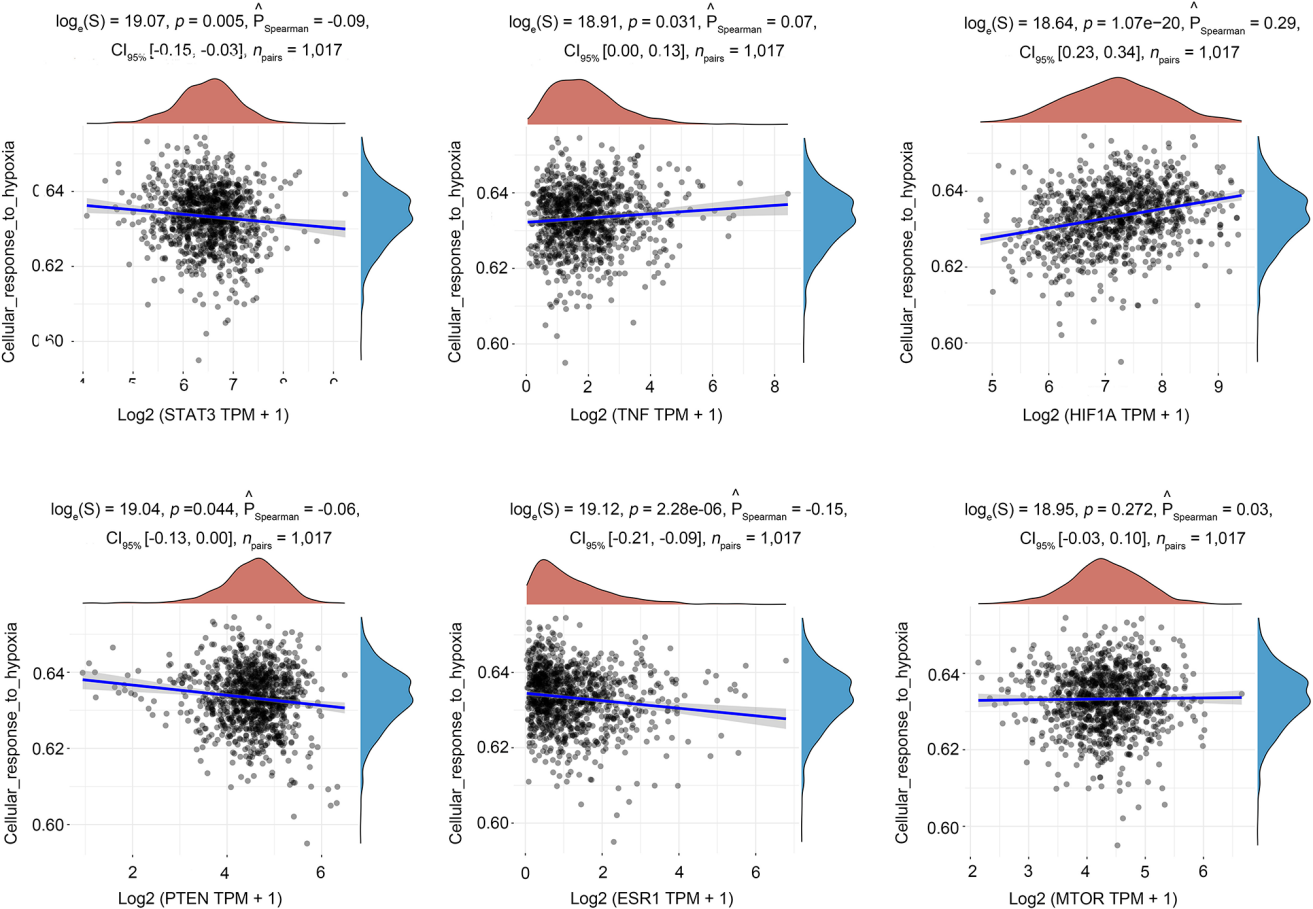
Correlation analysis of hub genes and cellular response to hypoxia pathway. * *p* < 0.05, ** *p* < 0.01, **** *p* < 0.0001

The Network Pharmacology results presented above indicate that HIF-1 A stands out as one of the top three hub genes, and the HIF-1 signaling pathway emerges prominently as one of the most enriched pathways. The widely acknowledged centrality of the HIF-1 pathway in orchestrating cellular responses under conditions of hypoxia is highlighted [31]. In light of this recognition, we postulate that the hypoxic response and the hypoxia-related HIF-1 signaling pathway constitute crucial molecular mechanisms through which Brevilin A exerts its anti-lung cancer effects. Our exploration aimed to investigate the correlations between the hub genes and the cellular response to the hypoxia pathway. Remarkably, our findings align perfectly with our initial assumptions. All of the top six hub genes (STAT3, TNF, HIF1A, PTEN, and ESR1), with the exception of MTOR, demonstrated significant associations with the cellular response to the hypoxia pathway (Fig. 8).

### Validation of molecular docking and molecular dynamics simulation

**Fig. 9:**
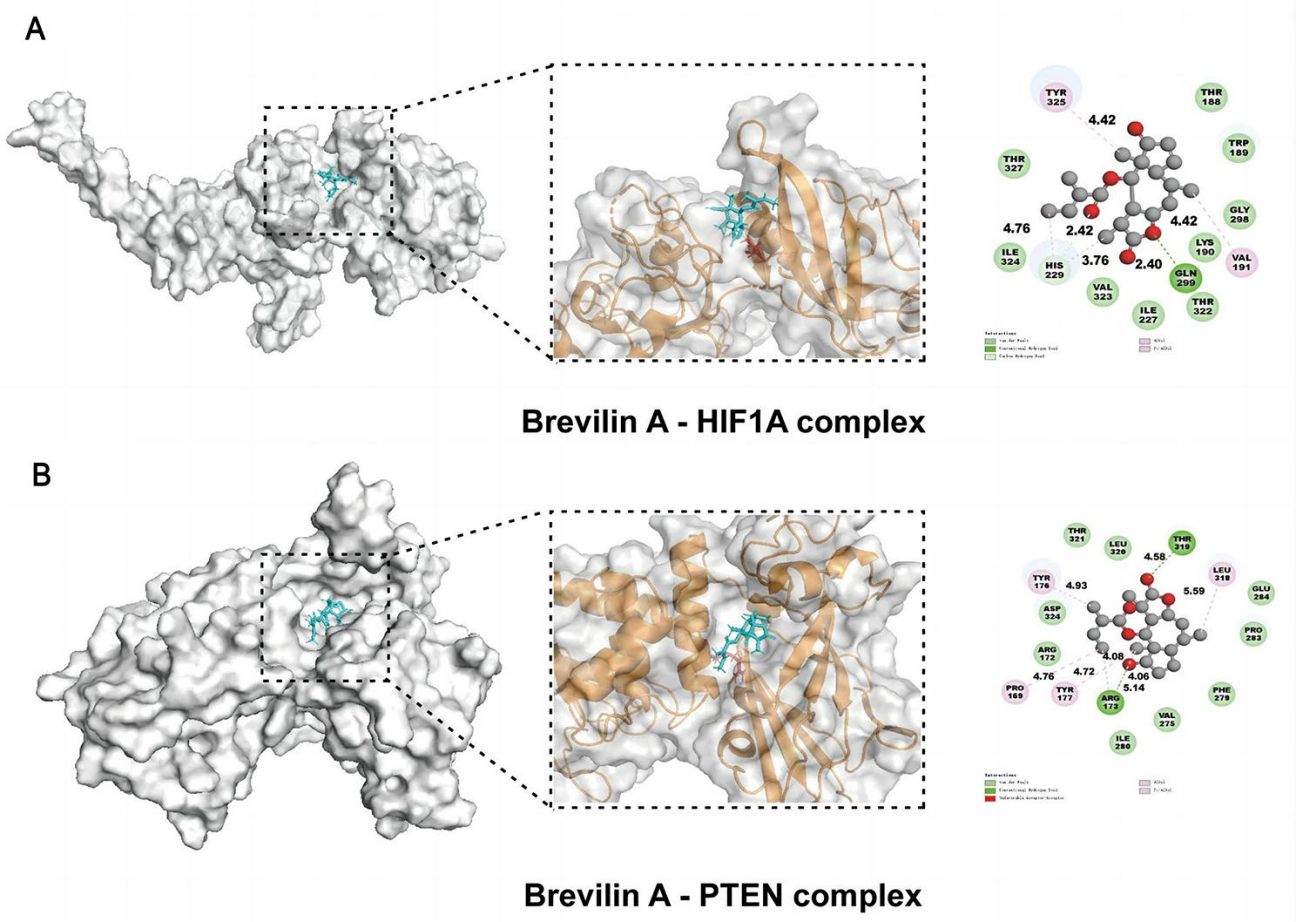
Molecular docking results. **(A)** The binding mode of Brevilin A-HIF1A complex. **(B)** The binding mode of Brevilin A-PTEN complex. The 3D visualization is on the left, and the 2D visualization is on the right

**Fig. 10:**
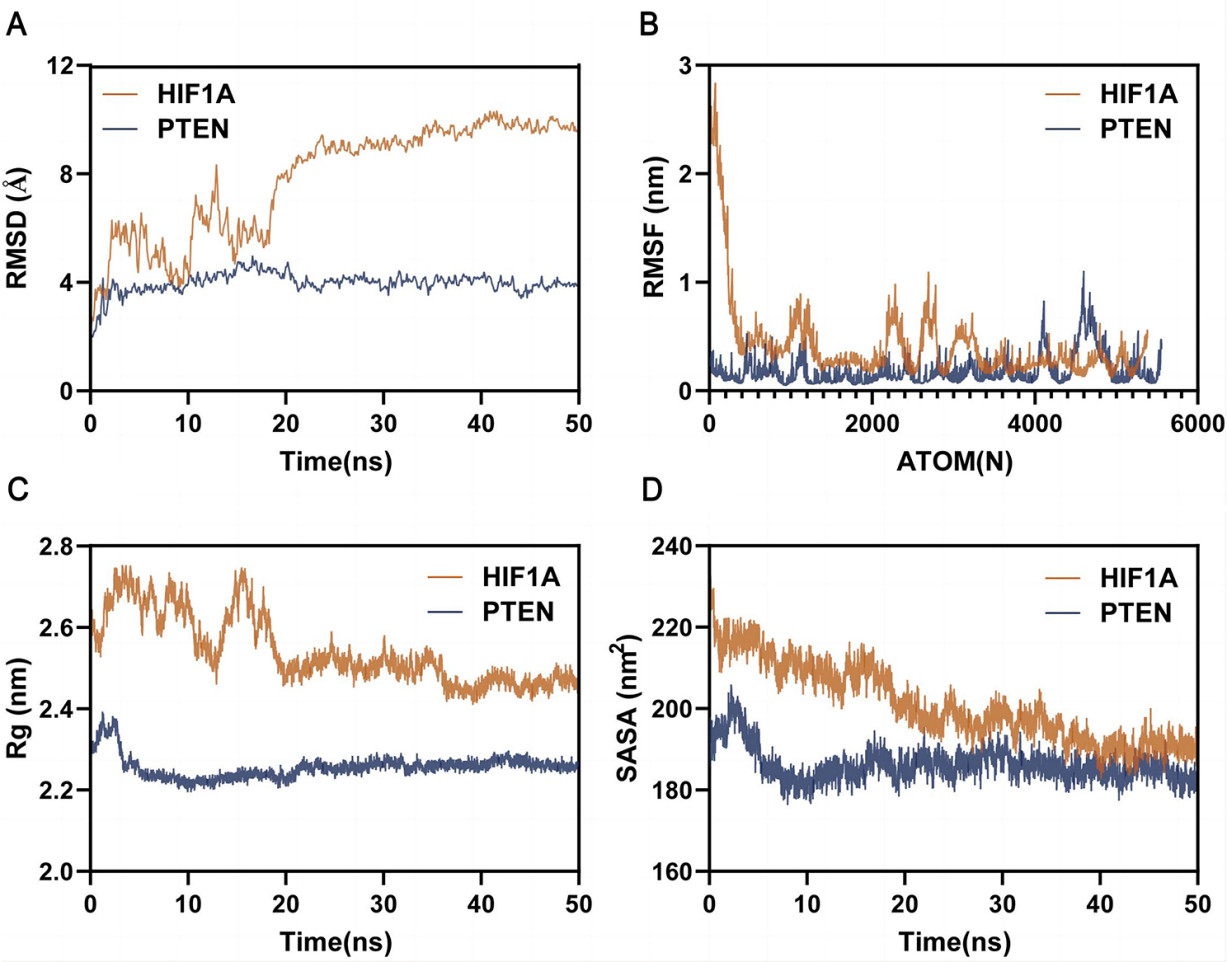
MD simulation analysis. **(A)** RMSD quantifying the deviation of complexes coordinates from the initial frame. **(B)** RMSF of individual protein atoms. **(C)** Rg for visualization of protein compactness. **(D)** SASA analysis of protein contact area with surrounding solvents

Building upon insights gleaned from prior investigations, the present study employed molecular docking and molecular dynamics simulation techniques to assess the binding mode and affinity of Brevilin A with HIF1A and PTEN targets. The outcomes of molecular docking are visually depicted in Fig. 9, encompassing both 2D and 3D representations. Additionally, Table 3 provides a succinct summary of fundamental information regarding binding mode and affinity. The results indicate robust bindings between Brevilin A and the respective targets, with binding affinities of -8.08 (HIF1A) and − 7.46 (PTEN). Following this, optimal conformations underwent a 50 ns molecular dynamics simulation to evaluate the stability of the binding. As illustrated in Fig. 10A, the RMSD of the complexes achieved stability at 20 ns, displaying only limited fluctuations, indicative of secure ligand binding to the target pockets. Subsequently, RMSF was computed to assess atom deviations within the proteins (Fig. 10B). The findings indicate that fluctuations primarily occurred at the terminals of the proteins, without compromising the integrity of the binding pocket.

**Table 3:** The binding pose and energy between Brevilin A and the targets

Target	(PDB ID)	Compound	amino acid interactions	Affinity (kcal/mol)
HIF1A	4ZPR	Brevilin A	VAL191, HIS229, GLN299, TYR325; THR188, TRP189, LYS190, ILE227, GLY298, THR322, VAL323, ILE324, THR327;	-8.08
PTEN	1D5R	Brevilin A	PRO169, ARG173, TYR176, TYR177, LEU318, THR319; ARG172, VAL275, PHE279, ILE280, PRO283, GLU284, LEU320, THR321, ASP324;	-7.46

Furthermore, the Rg value was employed to elucidate the conformational state of the proteins. As shown in Fig. 10C, proteins underwent a sequence of swelling and recovery prior to 20 ns, maintaining stability during the subsequent simulation period. An analysis of the SASA was conducted to assess the proteins’ interaction capability with surrounding solvents throughout the simulations (Fig. 10D). The findings demonstrated a reduction in SASA, suggesting a gradual enhancement in the binding affinity between the ligand and proteins [32]. The MM/PBSA approach, a predominant method for reassessing binding affinity, facilitated the calculation of the BFE between the ligand and protein [33]. Analysis of the results revealed that Brevilian-A-HIF1A and Brevilian-A-PTEN exhibited BFEs of -40.431 kJ/mol and − 80.088 kJ/mol, respectively (Table 4), indicative of a robust binding affinity.

**Table 4:** MMPBSA (kJ/mol) of protein-ligand complex

Energy type	HIF1A-Brevilin A	PTEN-Brevilin A
MM	-127.584	-150.515
PB	77.87	75.138
SA	-18.554	-23.575
Total Binding Energy	-68.268	-98.951
TΔS	27.837	18.863
Total Binding Free Energy	-40.431	-80.088

### Brevilin A-induced NSCLC cell death via targeting HIF-α pathway

**Fig. 11:**
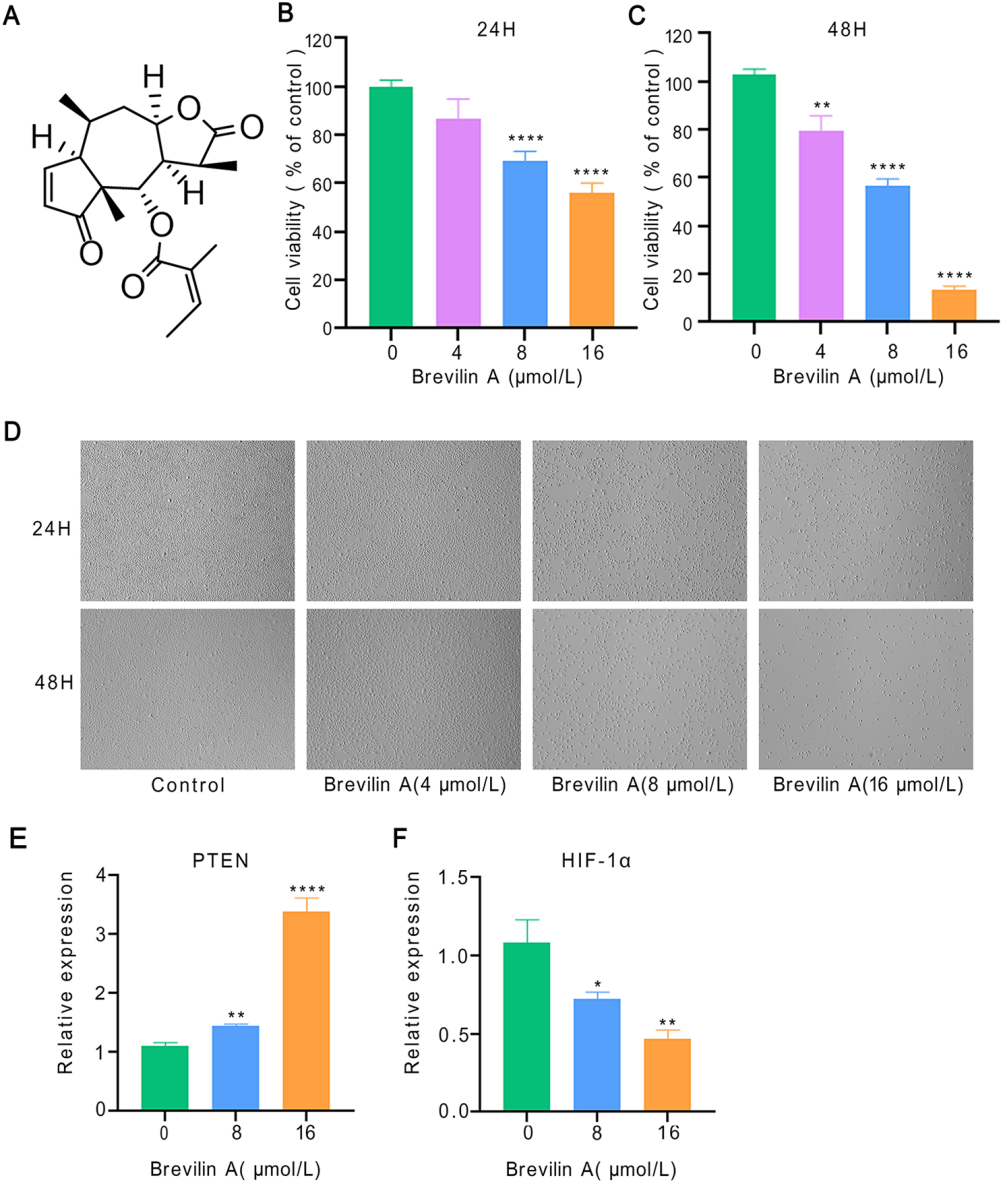
Brevilin A-induced A549 cell death via targeting HIF-1α pathway. **(A)** The chemical structure of the Brevilin A. CCK8 assay in A549 cells treated with Brevilin A for 24 h **(B)** and 48 h **(C)**. **(D)** Photographs of A549 cells after treatment for 24 h and 48 h. mRNA expression levels of PTEN **(E)** and HIF-1α **(F)** in A549 cells treated with Brevilin A for 24 h. ^*^p < 0.05, ^**^p < 0.01, ^****^p < 0.0001 versus vehicle control group

To assess the impact of Brevilin A (Fig. 11A) on lung carcinoma cells, we conducted a CCK-8 assay. Our results revealed that the administration of Brevilin A resulted in a dose- and time-dependent reduction in the viability of A549 cells (Fig. 11B, C). Following exposure to Brevilin A, there was a noticeable decrease in the quantity of adherent cells, accompanied by a morphological transformation characterized by a rounded appearance (Fig. 11D). These results unequivocally demonstrate the pronounced cytotoxicity of Brevilin A towards NSCLC cells, consistent with the outcomes derived from network pharmacology. To further validate the outcomes obtained through network pharmacology, we examined the mRNA expression level of HIF-1α in A549 cells. Remarkably, Brevilin A treatment significantly reduced HIF-1α expression in a dose-dependent manner (*p* < 0.05) (Fig. 11F). Additionally, exposure to Brevilin A led to a significant increase in PTEN mRNA levels (*p* < 0.05) (Fig. 11E). These findings indicate that Brevilin A potentially induced NSCLC cell death by targeting the HIF-α pathway, aligning with the network pharmacology results.

## Disscussion

Sesquiterpene lactones, derived from plants, are widely employed in TCM for their anti-inflammatory and anticancer properties [34]. These compounds exhibit reactivity with functional groups, notably the thiol group on proteins and enzymes. They demonstrate selectivity towards tumor and cancer stem cells by targeting specific signaling pathways, making them noteworthy agents in cancer clinical trials [35, 36]. Previous studies from our research group have established the neuroprotective effects of Brevilin A against lipopolysaccharide-induced neuroinflammation both in vitro and in vivo. In the present study, we elucidate the therapeutic efficacy of Brevilin A in the context of lung cancer, operating through multi-target, multi-biological processes, and multi-pathway mechanisms.

Our results highlight STAT3, TNF, HIF1A, PTEN, ESR1, and MTOR as potential therapeutic targets for Brevilin A’s anticancer activity. STAT3, a transcription factor integral to diverse biological processes, including cell proliferation, survival, differentiation, and angiogenesis [37], has been implicated in various human cancers, such as head and neck tumors, cervical cancer, gastric carcinoma, and colon cancer [38–41]. Notably, exosome-mediated transfer of specific microRNAs has been associated with the activation of STAT3 signaling-induced epithelial-mesenchymal transition in lung cancer cells [42]. TNF-α, a member of the tumor necrosis factor superfamily, exhibits a spectrum of biological activities [43] and has been implicated in numerous human cancers, influencing processes such as growth, invasion, and metastasis [44, 45]. In NSCLC patients, elevated levels of IL-1, IL-6, and TNF-α have been linked to cancer pain and prognosis [46]. HIF (hypoxia-inducible factor) [47], a transcription factor crucial for tumor angiogenesis, cell survival, proliferation, apoptosis, metastasis, infiltration, and metabolism [48], plays a pivotal role in promoting lung cancer cell proliferation under conditions of chronic intermittent hypoxia [49]. Studies also demonstrate that certain formulations in TCM can inhibit NSCLC cell proliferation by downregulating HIF-1α expression [50]. Phosphatase and tensin homolog deleted on chromosome ten (PTEN), encoding the classical PTEN protein with phosphatase activity, acts as a tumor suppressor by antagonizing the activity of tyrosine kinases and other phosphorylases. Meta-analyses indicate a correlation between PTEN and poor prognosis in lung cancer [51], and clinical studies confirm abnormal expression of EGFR, TGF-α, P-AKT, and PTEN in NSCLC patients [52], potentially contributing to NSCLC pathogenesis. In the present study, we utilized SwissDock website to investigate Brevilin A’s potential binding sites with these proteins, revealing robust binding activity. GSEA analysis further validated the strong association of these targets with lung cancer.

The GO and KEGG analyses revealed 2893 enriched GO terms and 157 enriched KEGG pathways, encompassing notable pathways such as the PI3K-Akt signaling pathway, FoxO signaling pathway, and HIF-1 signaling pathway. The PI3K-Akt signaling pathway, governing various cellular functions including growth, differentiation, proliferation, survival, motility, invasion, and intracellular trafficking, plays a pivotal role in tumorigenesis [53]. Studies have reported the induction of apoptosis and inhibition of invasion in NSCLC through the PI3K/Akt/mTOR signaling pathway by compounds like Aloperine [54]. Additionally, CAF-derived exosomes have been identified to promote NSCLC cellular proliferation and chemoresistance through regulation of the PTEN/PI3K-AKT signaling axis [55]. The FOXO signaling pathway, triggered by the PI3K/AKT pathway, is instrumental in mediating cell proliferation, differentiation, and tumorigenesis [56, 57]. Inhibition of CCCTC-binding factor (CTCF) has been shown to regulate the FoxO signaling pathway, impeding tumor growth in vivo [58]. Notably, our study is the first to unveil that Brevilin A exerts anti-lung cancer effects by targeting the HIF-1 signaling pathway. Gene-pathway correlation analysis further revealed significant associations between most hub genes and the cellular response to hypoxia pathway. Hypoxia, influencing tumor signaling pathways through hypoxia-inducible factors (HIFs) and reducing free radical production, holds significance in tumor progression. Studies have demonstrated the role of hypoxia in activating EGFR and inducing resistance to gefitinib in EGFR-mutant non-small cell lung cancer [59]. Silencing HIF-1α expression has been shown to significantly reduce the invasive ability of lung cancer cells under hypoxic conditions [60]. Molecular docking analysis and molecular dynamics simulation affirmed the robust interaction of Brevilin A with HIF1A and PTEN, respectively. In vitro experiments demonstrated that Brevilin A induces dose- and time-dependent cell death in A549 cells, concomitant with decreased HIF-1α mRNA expression and increased PTEN mRNA levels. These results suggest the potential of the HIF-1 signaling pathway as a therapeutic target for Brevilin A in lung cancer treatment.

In summary, our study delineated the core targets and key pathways of Brevilin A in lung cancer through an integrated approach involving network pharmacology, molecular docking analysis, and experimental validation. The therapeutic effects of Brevilin A in lung cancer were demonstrated to involve a multi-target, multi-biological process, and multi-pathway mechanism, with noteworthy inhibition of the HIF-1 signaling pathway. These results lay a theoretical foundation for the prospective clinical application of Brevilin A. Nevertheless, it is imperative to acknowledge certain limitations in this study. Firstly, the utilization of more comprehensive databases would enhance the reliability of results. Secondly, further experimental validations are imperative to consolidate the findings derived from network pharmacology. Therefore, additional investigations are warranted to unravel the anti-lung cancer molecular mechanisms of Brevilin A.

## Data Availability

The datasets used and/or analyzed in the current study are available from the corresponding author upon reasonable request.

## Abbreviations

TCM: Traditional Chinese medicine
*C. minima*: Centipeda minima (L.)A.Br.et Aschers
PPI: Protein-protein interaction
GO: Gene Ontology
KEGG: Kyoto Encyclopedia of Genes and Genomes
GSEA: Enrichment analysis
NSCLC: Non-small cell lung cancer
DEGs: Gene differential expressions
MCC: Maximum Correlation Clique
CC: Cellular component
BP: Biological process
MF: Molecular function
RESP: Restrained electrostatic potential
RMSD: Root mean square deviation
RMSF: Root mean square fluctuation
Rg: Radius of gyration
SASA: Solvent accessible surface area
BFE: Binding free energies
MM/PBSA: Molecular mechanics/Poisson-Boltzmann surface area
IE: Interaction entropy
